# Understanding the impact of pediatric single large‐scale mtDNA deletion syndromes on caregivers: Burdens and challenges

**DOI:** 10.1002/jmd2.12385

**Published:** 2023-08-03

**Authors:** McKenzie Chappell, Sumit Parikh, Elizabeth Reynolds

**Affiliations:** ^1^ Case Western Reserve University School of Medicine Cleveland Ohio USA; ^2^ Mitochondrial Medicine Center, Neurosciences Institute Cleveland Ohio USA; ^3^ The Champ Foundation Durham North Carolina USA

**Keywords:** caregiver burden, Kearns Sayre syndrome, Pearson syndrome, mtDNA, rare disease registry, single large‐scale mitochondrial deletion syndromes

## Abstract

Single large‐scale mitochondrial deletion syndromes (SLSMDS) are ultra‐rare, progressive multi‐system diseases that make children largely dependent on their caregivers for both medical and non‐medical needs. Yet, few studies have examined the burden felt among caregivers. As part of a larger research study, 42 caregivers of children with SLSMDS completed two surveys to assess caregiver burden. The Mitochondrial Care Network Patient Needs Survey (MCN‐PNS) is a novel assessment that examines the logistical, time, and financial costs experienced by caregivers of children with SLSMDS. The Zarit Burden Interview (ZBI‐22) is a validated assessment that examines caregivers’ psychological health. Results demonstrate the unique burden experienced by caregivers of children with SLSMDS. One notable finding was the high psychological burden. Nearly 90% of caregivers experience psychological burden, with 20% of caregivers at risk for anxiety and depression. Caregivers were primarily concerned about what the future held for their child. Additional burdens included the time required to coordinate the child's healthcare visits and financial strains. Caregivers reported minimal delays in establishing care with a mitochondrial care specialist and felt confident in their understanding of their child's disease and treatment(s). Overall, there is a need for expanded logistical, financial, and psychological support from mitochondrial disease centers and advocacy groups for caregivers of children with SLSMDS.

**Synopsis**

Caregivers of children with single large‐scale mtDNA deletion syndromes (SLSMDS) experience unique financial, logistical, and psychological burdens.

## 1 INTRODUCTION

Single‐large scale mitochondrial deletion syndromes (SLSMDS) are a spectrum of rare mitochondrial diseases including Pearson syndrome (OMIM: 557000), Kearns‐Sayre syndrome (KSS; OMIM: 530000), chronic progressive external ophthalmoplegia (CPEO; OMIM: 157640, 610131) and SLSMDS not otherwise specified (NOS), caused by a spontaneous deletion in mitochondrial DNA (mtDNA).^1^ The prevalence of SLSMDS is estimated to be 1:1 000 000 and presents as a progressive multi‐system disease with a broad array of neural, hematologic, and gastrointestinal symptoms.^1^, ^2^ Advances in the hematologic management of Pearson syndrome have been successfully improving prognosis and longevity,^3^ however the lifetime outlook remains bleak, with most patients requiring intensive symptom management and transfusions.^1^ KSS is characterized by external ophthalmoplegia and pigmentary retinopathy and high cerebrospinal fluid (CSF) protein content, cardiac conduction block, and/or ataxia.^1^, ^2^ Children can initially present with Pearson syndrome and then transition to KSS. Both phenotypes are associated with high morbidity, and neither has effective treatments or cures.^2^, ^3^

Having a child with a complex, fatal illness is devastating for all members of the family and drastically impacts daily functioning.^4^ Mothers, fathers, or other guardians (herein called “caregivers” for clarity) often experience great logistical and financial burden as they reduce their time at work to coordinate office visits, hospital stays, and travel accommodations.^4^, ^5^, ^6^ One study examining the caregiver burden among parents of children with Dravet syndrome found that the caregiver's overall reduction in quality of life was, in part, due to their reduced workforce participation and increased time coordinating care and traveling to their child's appointments.^7^, ^8^ Additionally, numerous studies have found that increased caregiver burden significantly damages the physical and psychological health of parents^9^; placing them at a higher risk of anxiety, depression, and chronic fatigue.^10^, ^11^ Causes of poor caregiver health outcomes are believed to be multifactorial; the extent to which caregivers must provide medical care for their child and live with the uncertainty of their future have both been implicated as negative stressors.^12^, ^13^, ^14^ These outcomes are especially prevalent among caregivers of children with chronic neurological diseases and rare diseases.^7^, ^15^ One possible explanation for this is the degenerative nature of pediatric neurologic disease; in which the healthcare team and caregivers must balance slowing disease progression and maintaining the child's quality of life.^16^ Moreover, neurologic and rare diseases tend to produce severe symptoms that often require intensive therapeutic interventions that are not used in more common diseases.^12^ This can make caregivers feel isolated in their experience and subject to scrutiny from friends and family; further amplifying feelings of anxiety and depression.^9^, ^14^, ^17^

Despite the progressive nature of pediatric SLSMDS, few studies have examined the burden felt among caregivers although it is important to have quality indicators of burden for future clinical trials.^18^ In this study, we examined burden and psychological health among caregivers whose children are diagnosed with SLSMDS.

## 2 MATERIALS AND METHODS

### 2.1 CFR and registry procedures

Data from the present study was obtained from the Champ Foundation Registry (CFR). The CFR is a data registry of individuals with SLSMDS, established in 2020 and financially supported by The Champ Foundation. The registry was developed to enhance the understanding of SLSMDS through patient data and includes patient reported outcomes (PROs), genetic records, natural history study data, electronic health record data, and biospecimen data. Caregivers, defined in the CFR as “parents or legal guardians,” or individuals with a genetic or clinical diagnosis of SLSMDS are eligible to participate. All registry procedures including participant recruiting, surveys, data sharing were approved by Castle IRB (IORG #: IORG0010151) in July 2020. Participants were recruited in a number of ways including social media and direct‐to‐patient outreach by The Champ Foundation. Participants first create an online patient profile and provide informed consent agreeing to participate in the research study. Finally, participants can complete PRO surveys that are available on their dashboard.

### 2.2 Participants

There are over 100 participants in the CFR, spanning all clinical presentations of SLSMDS. To be included in the present study's analyses, participants must have (1) had a caregiver register the affected individual with a SLSMDS (i.e., not self‐registered), and (2) completed at least one of two caregiver burden scales (*n* = 42).

### 2.3 Instruments

Two scales were utilized to assess caregiver burden: the novel MCN‐PNS and the ZBI‐22.

#### 2.3.1 MCN‐PNS

This survey was developed to better understand the uniqueness of the mitochondrial disease patient community. The survey was developed in 2019 by the Mitochondrial Care Network (MCN). The MCN represents a group of physicians at medical centers across the country that have expertise and experience in providing coordinated, multidisciplinary care for patients with genetic mitochondrial disease.^19^ The complete MCN‐PNS is 46 items and includes multiple choice questions and open‐ended text responses. The 26 items utilized in analyses focused on logistical (e.g., establishing, coordinating, and understanding care) and cost (e.g., time, financial) burdens. The complete MCN‐PNS is in Table S1.

#### 2.3.2 ZBI‐22

This survey is a validated, 22‐item survey with five response options (ZBI‐22). The purpose of the ZBI‐22 is to measure a caregiver's perceived burden and items focus on psychological burdens such as the caregiver's health, social life, and the relationship between the caregiver and the patient.^20^ The 5‐point Likert scale response options include: 0 = never, 1 = rarely, 2 = sometimes, 3 = quite frequently, 4 = nearly always. All items can be summed to calculate a total score, ranging from 0 (lowest burden) to 88 (highest burden). Previous research suggests a total score of 21 is a burden cut point^8^, ^20^, ^21^ and a total score of 48 identifies caregivers at risk of anxiety and depression.^21^ The complete ZBI‐22 is in Table S2.

#### 2.3.3 Demographics

In addition to completing the caregiver burden surveys, caregivers reported child and family demographic characteristics upon CFR enrollment: SLSMDS diagnosis, child date of birth, sex, country, family income, and the registering person's relationship to the child (Table 1).

**TABLE 1:** Demographic and clinical data of caregivers and their child with SLSMDS (*N* = 42).

Survey item	Response options	*n*	%
Sex	Female	17	40%
	Male	25	60%
Country	United States	25	60%
	Other	17	40%
Diagnosis	Pearson syndrome	28	67%
	KSS	8	19%
	CPEO	2	5%
	SLSMDS NOS	4	10%
Family income	Decline to respond	6	14%
	Less than $20 000	4	10%
	$20 000–$49 000	7	17%
	$50 000–$99 000	10	24%
	$100 000 or greater	15	36%
Relationship to affected individual	Mother	39	93%
	Father	3	7%

#### 2.3.4 Time frame for survey completion

The CFR launched in August 2020. The ZBI‐22 first became available to all living CFR participants in October 2020. Dates of completion range from October 16, 2020 to May 18, 2022. The MCN‐PNS first became available to all living CFR participants in May 2021. Dates of completion ranged from May 1, 2021 to February 2, 2023. On average, there was a 5.6‐month difference between completing the MCN‐PNS and ZBI‐22 survey. Seven participants completed the MCN‐PNS and ZBI‐22 on the same day.

### 2.4 Analysis

All analyses were conducted in SAS 9.4. Descriptive statistics including percentages, means, and standard deviations were used to describe demographic and clinical data of caregivers and their children with SLSMDS. Percentages were used to describe survey results from the MCN‐PNS and ZBI‐22 survey.

## 3 RESULTS

### 3.1 Child and family demographic characteristics

Data from 42 caregivers were utilized in the present study. Caregivers reported their child's SLSMDS phenotype, including Pearson syndrome (67%, *n* = 28), KSS (19%, *n* = 8), CPEO (5%, *n* = 2), and SLSMDS NOS (10%, *n =* 4). The average age of the affected individuals at the time of survey completion was 8.59 (0–21) years. Sixty percent were male (*n* = 25) and 60% were from the United States (*n* = 25). International participants were from Africa (*n =* 1), Asia (*n* = 3), Australia *(n* = 2), Europe (*n* = 9), North America (*n* = 1) and South America (*n* = 1). Over 35% of families had incomes of $100 000 or greater. Most commonly, the mother (93%, *n* = 39) was the caregiver responsible for creating the CFR profile and completing surveys (Table 1).

### 3.2 Results from the MCN‐PNS

MCN‐PNS data from 38 caregivers are included in the present analyses. We categorized results from the MCN‐PNS in Table 2 in terms of logistical burden (establishing care, coordinating care, gathering and understanding medical information) and cost burden (time cost and financial cost). Additionally, a final survey question on the MCN‐PNS asked caregivers about overall barriers or obstacles to care. Findings as they relate to logistical burden and cost burden are described below. Full results from the MCN‐PNS can be found in Table S1.

**TABLE 2:** Caregiver responses related to logistical and cost burden from the Mitochondrial Care Network Patient Needs Survey (MCN‐PNS; *N* = 38).

Survey item	Response options	*n*	%
Logistical burdens			
Establishing care			
How long did it take for you to be referred to a mitochondrial specialist after mitochondrial disease was suspected?	Less than 6 months	24	63%
	6–12 months	4	11%
	1–2 years	1	3%
	2–3 years	2	5%
	Other	7	18%
If you were not immediately referred to a mitochondrial specialist after suspected mitochondrial disease diagnosis, please describe why there was a delay (*select all*)	PCP unaware of mitochondrial disease center	0	0%
	No local mitochondrial disease center available	11	29%
	Cost/insurance coverage	5	13%
	Other	5	12%
How long did you have to wait between scheduling the appointment and being seen as a new patient at the mitochondrial center?	0–2 weeks	10	33%
	2–4 weeks	3	10%
	1–2 months	6	20%
	3–5 months	5	16%
	Greater than 6 months	6	20%
Have you experienced a delay in scheduling a follow‐up appointment with your mitochondrial specialist?	No delay	26	76%
	1 week	0	0%
	Less than a month	1	3%
	1–3 months	1	3%
	Greater than 3 months	6	18%
Coordinating care			
How many medical centers/locations do you have to attend to receive all your mitochondrial specialty care?	1	18	53%
	2–3	11	32%
	4–5	4	12%
	Greater than 5	1	3%
Does your mitochondrial care team communicate with your primary care provider and other specialists (share clinic notes, discuss care/recommendations, etc.)?	Yes	27	79%
	No	3	9%
	I do not know	4	12%
Does your mitochondrial care team address non‐medical concerns associated with mitochondrial disease such as social work or psychiatric care?	Yes	16	47%
	No	14	41%
	I do not know	4	12%
When discharged from the hospital, do you feel that you have adequate coordination of your medical care including appropriate support services and knowing who to contact with any issues of concerns?	Yes	26	79%
	No	5	15%
	I do not know	2	6%
Do you have a point person/care coordinator through your mitochondrial center?	Yes	19	56%
	No	13	38%
	I do not know	2	6%
Gathering and understanding medical information			
Prior to your appointment—were you provided enough information so that you could prepare for your visit, know exactly what to expect at the appointment, and prepare your questions?	Yes	20	59%
	No	8	24%
	I do not know	6	18%
On a scale of 1–5, how easy is it to understand the medical information your mitochondrial specialists share with you?	(1) I understand all the information	15	44%
	(2) I understand most of the information	15	44%
	(3) I understand some of the information	3	9%
	(4) I do not understand most of the information	1	3%
	(5) I do not understand any of the information	0	0%
Do you leave your visit with a care plan and next steps from your mitochondrial specialists including how to ask follow‐up questions?	Yes	29	85%
	No	5	15%
	I do not know	0	0%
Do you feel like there is an opportunity to ask all of your questions at each mitochondrial center visit?	Yes	29	85%
	No	4	12%
	I do not know	1	3%
Did the mitochondrial clinic explain clearly to what extent they can be involved in your care and what are their limitations?	Yes	19	59%
	No	9	28%
	I do not know	4	13%
Cost burdens			
Time cost			
How many times per year do you visit your mitochondrial center?	Infrequently, less than once per year	3	10%
	Once per year	8	26%
	2–3 times per year	16	52%
	4–5 times per year	0	0%
	>5 times per year	4	13%
How long does it take you to arrive at the mitochondrial center from your home (this includes total driving, flying or other transportation time)?	Less than 1 hour	8	24%
	1–3 hours	9	27%
	3–5 hours	8	24%
	5–10 hours	6	18%
	Greater than 10 hours	2	6%
On average, how long does it take to complete all of your appointments at the mitochondrial center?	1 day	19	58%
	2–3 days	12	36%
	4–5 days	2	6%
	Greater than 1 week	0	0%
How many hospitalizations or emergency room visits have you had in the past year?	0	7	21%
	1–2	11	32%
	3–4	13	38%
	5_10	3	9%
	Greater than 10	0	0%
Financial cost			
How do you travel to the mitochondrial center? (*select all*)	Car	26	68%
	Airplane	9	24%
	Shuttle service	1	3%
	Bus/train	4	11%
	Other	0	0%
How much do you spend per night for accommodations?	Not applicable	14	47%
	Less than $50	0	0%
	$50–$100	3	10%
	$100–150	9	30%
	Greater than $150	4	13%
What is the average cost per day for parking onsite when attending mitochondrial center?	Not applicable‐ parking is free	11	32%
	Less than $5.00	4	12%
	$5.00–$10.00	6	18%
	$10.00–$15.00	4	12%
	Greater than $15.00	9	26%
Does your mitochondrial center offer a meal voucher?	Yes	5	15%
	No	21	62%
	I do not know	8	24%
How much money do you spend on meals per day per person when you attend mitochondrial center?	I do not purchase food while attending clinic	8	24%
	Less than $20	6	18%
	$20–$40	12	35%
	$40–$60	8	24%
	Greater than $60	0	0%
Is the mitochondrial center you attend considered in‐network with your insurance company?	Yes	19	58%
	No	10	30%
	I do not know	4	12%
Are physician visits a burden on family finances due to copays, parking, food, gas, etc.?	Yes	15	44%
	No	15	44%
	I do not know	4	12%
Overall barriers			
Please select one or more barriers/obstacles that are present in your care for mitochondrial disease (select all)	No barriers	4	11%
	Distance to travel to mitochondrial center	17	45%
	Transportation	8	21%
	Lodging	8	21%
	Childcare	6	16%
	Insurance coverage/cost of appointments	10	26%
	Loss of work/wages	7	18%
	Lack of referral	2	5%
	Lack of necessary medical specialties/services	8	21%
	Lack of care coordination for multisystem disease	10	26%
	Lack of continuity of care between multiple medical specialists	8	21%
	Lack of non‐medical support (social, psychological, financial)	9	24%
	Other	2	5%

### 3.3 Logistical burdens

#### 3.3.1 Establishing care

Survey items related to establishing care include time it took to be referred to a mitochondrial specialist, reasons for delayed referral, wait time between scheduling appointments and being seen, and time delay in follow‐up care (Table 2). To highlight one example, the majority of caregivers (63%; *n* = 24) reported being referred to a mitochondrial specialist in less than 6 months. If there was a delay in establishing care, most caregivers said the delay was because there was no local mitochondrial disease center available (29%; *n* = 11).

#### 3.3.2 Coordinating care

Survey items related to coordinating care include number of medical centers attended, mitochondrial care team communication with primary care provider, mitochondrial care team addressing non‐medical concerns, whether participants feel they have adequate coordination of medical care, and whether they have a point person/care coordinator (Table 2). To highlight one example, most caregivers (56%; *n* = 19) also said they have a specific point person/care coordinator through their mitochondrial center.

Caregivers reported overall care burdens related to coordinating care costs, including “lack of care coordination for multisystemic disease” (26%; *n* = 10), “lack of continuity of care between multiple medical specialists” (21%; *n* = 8), and “lack of necessary medical specialties/services” (21%; *n* = 8; Table 2).

#### 3.3.3 Gathering and understanding medical information

Survey items related to gathering and understanding medical information include feeling prepared for appointments, ability to understand medical information, whether they leave appointments with care plans and next steps, whether they feel all their questions are answered, and whether the clinic clearly explained extent they can be involved in care (Table 2). To highlight one example, while many caregivers (59%; *n* = 20) said they had enough information to prepare for a visit, knew exactly what to expect at their appointment, and prepare questions, there was nearly a quarter of caregivers (24%; *n* = 8) who reported they did not feel adequately prepared.

### 3.4 Cost burdens

#### 3.4.1 Time cost

Survey items related to time cost include times per year families visited mitochondrial center, time it takes to travel, time it takes to compete appointments, and number of hospitalizations or emergency room visits (Table 2). To highlight one example, most caregivers (65%; *n* = 20) reported visiting their mitochondrial center two or more times per year.

When describing overall burdens, caregivers said: “childcare” (15.79%; *n* = 6) was an obstacle to care (Table 2).

#### 3.4.2 Financial cost

Survey items related to financial cost include types of travel to mitochondrial centers, cost per night for accommodations, cost per day for parking, whether their center has meal vouchers, cost for meals, whether their center is in‐network for insurance, and whether physician visits are a burden on family finances (Table 2). To highlight one example, when asked whether physician visits are a burden on family finances, 44% responded yes (*n* = 15).

Caregivers reported overall care burdens related to financial costs, including “insurance coverage/cost of appointments” (26%; *n* = 10), “transportation” (21%; *n* = 8), “lodging” (21%; *n* = 8), and “loss of work/wages” (18%; *n* = 7; Table 2).

### 3.5 Results from the ZBI‐22

ZBI‐22 data from 33 caregivers are included in the present analyses. We highlight specific results demonstrating psychological burden below and in Table 3. Full results from the ZBI‐22 can be found in Table S2.

**TABLE 3:** Frequency caregivers endorsed “quite frequently” or “nearly always” on the Zarit Burden Interview (ZBI‐22; *N* = 33).

Item	Endorsed “quite frequently” or “nearly always”	
	*N*	%
Are you afraid what the future holds for the affected individual?	28	85%
Do you feel the affected individual is dependent on you?	24	75%
Do you feel stressed between caring for the affected individual and trying to meet other responsibilities for your family or work?	18	55%
Do you feel that because of the time you spend with the affected individual that you do not have enough time for yourself?	12	36%
Do you feel strained when you are around the affected individual?	11	33%
Do you feel that you do not have enough money to take care of the affected individual in addition to the rest of your expenses?	11	33%
Overall, how burdened do you feel in caring for the affected individual?	11	33%
Do you feel like you should be doing more for the affected individual?	9	29%
Do you feel that your social life has suffered because you are caring for the affected individual?	9	27%
Do you feel like you have lost control of your life since the affected individual's illness?	7	21%
Do you feel uncertain about what to do about the affected individual?	7	21%
Do you feel like you could do a better job in caring for the affected individual?	7	21%
Do you feel that you do not have as much privacy as you would like because of the affected individual?	6	18%
Do you feel that the affected individual currently affects your relationship with other family members or friends in a negative way?	5	15%
Do you feel that the affected individual seems to expect you to take care of him/her as if you were the only one he/she could depend on?	5	15%
Do you feel that the affected individual asks for more help than he/she needs?	5	15%
Do you feel embarrassed over the affected individual's behavior?	2	6%
Do you feel angry when you are around the affected individual?	1	3%
Do you feel uncomfortable about having friends over because of the affected individual?	1	3%
Do you wish you could leave the care of your affected individual to someone else?	1	3%
Do you feel that you will be unable to take care of the affected individual much longer?	0	0%

*Note*: Items are listed starting from the highest percentage of caregivers who endorsed these items, representing higher burden.

### 3.6 Psychological burdens

Caregivers’ mean total score for the ZBI‐22 was 33 (SD = 12.27; range 11–62). Specifically, 88% (*n* = 29) of caregivers’ mean total scores were higher than the 21‐point cutoff. Nearly 20% (*n* = 6) of caregivers’ total scores were 48 or higher, identifying a subset of parents at risk for anxiety and depression.

Table 3 lists the items where caregivers most frequently endorsed “quite frequently” or “nearly always.” Items are listed starting from the highest percentage of caregivers who endorsed these items, representing a higher burden. The highest frequency of caregivers (85%; *n* = 28) reported “quite frequently” or “nearly always” when asked if they were afraid for what the future holds for the affected individual.

## 4 DISCUSSION

In the present study, we describe the unique burden felt by caregivers of children with SLSMDS using both a novel and standardized caregiver burden scale. The results show that caregivers, most of whom are mothers, experienced a high psychological burden, moderate‐to‐high cost burden, and low‐to‐moderate logistical burden.

Our most notable finding was the high psychological burden for caregivers of children with SLSMDS. Results from the ZBI‐22 suggest that nearly 90% of caregivers experience psychological burden, with 20% of caregivers at risk for anxiety and depression. This may be a result of the expected poor prognosis for children with SLSMDS. Unlike other pediatric disorders, there is no treatment for SLSMDS and no active clinical trials.^1^ The item most endorsed as “quite frequently” or “nearly always” was being “afraid for what the future holds for the affected individuals.” The high psychological burden may also be reflective of dependency. Similar to other children with complex medical disorders, children with SLSMDS are very dependent on their caregiver; many require multiple medications per day, have feeding tubes or insulin pumps, and require frequent dialysis or blood transfusions.^2^ To take care of their children, caregivers may have less time for themselves which has been shown to amplify feelings of depression, fear, and hopelessness.^16^ More so, other studies have suggested that caregivers’ burden may be a result of isolation and limited information caused by the rarity of their child's disorder.^8^ Lastly, previous research on caregiver burden for mitochondrial disease suggests that some psychological burden, particularly for mothers, may be related to the long‐held idea that mitochondrial diseases are caused by inherited defects in the maternal mtDNA. Mothers of children with SLSMDS may feel excessive guilt and responsibility for their child's disease, even when it was not inherited.^22^

The cost burden, in terms of time and financial cost, was moderate‐to‐high for caregivers of children with SLSMDS. Caregivers reported multiple trips to mitochondrial centers per year, frequent hospitalizations, the need to travel multiple hours to visit their center, and visits often lasting more than 1 day. Many families traveled by airplane, spent over $100 per night on hotel rooms, and paid over $15 per day in hospital parking fees. Nearly half of families reported that physician visits are a burden on family finances. The high financial burden placed on caregivers of children with chronic, rare diseases can be attributed to socio‐economic and disease‐related factors. The socio‐economic status of the caregiver largely determines the families access to care and support systems; with low‐income families experiencing the greatest financial strain.^23^ Rare diseases require an extensive multidisciplinary team, at‐home medical equipment, and newer medical therapies that can exhaust or be denied by medical insurance; placing all of the financial burden on the caregiver.^6^, ^9^, ^11^, ^13^, ^14^, ^24^

Most caregivers described relatively low‐to‐moderate logistical burdens. The majority of caregivers were referred to a mitochondrial specialist in under 6 months and the majority said there was no delay in scheduling follow‐up care. Indeed, the MCN aims to establish care with newly diagnosed patients and their families within 2 weeks and offers a virtual second opinion consultation service for families who may not have a mitochondrial care center in their state. Nonetheless, a subset of families described a greater burden while establishing care; for example, nearly 20% of caregivers said they needed to wait longer than 6 months to have their new patient appointment and longer than 3 months to schedule a follow‐up appointment. Obtaining a rare disease diagnosis can be an arduous process because of symptom heterogeneity and lack of physician awareness.^14^, ^25^, ^26^ Physicians are trained to think systematically when approaching an undifferentiated patient; which can unintentionally prolong the diagnostic process and limit understanding of rare diseases.

While most caregivers reported being prepared and knowing what to expect at appointments, nearly a quarter of caregivers reported feeling inadequately prepared. Caregivers report burdens related to care coordination, which is unsurprising given the complex, multi‐systemic nature of SLSMDS. Many families visit multiple mitochondrial centers. This has the potential for improvement as the use of electronic medical records becomes the standard of care; which can streamline communication between members of the healthcare team and the patient's caregiver.^27^ Nevertheless, there is significant strain placed on caregivers of children with rare diseases to plan numerous specialist visits. In addition to coordinating these visits, they must consider all related costs, time spent traveling and out of work, and any mobility assistance their child may need.^6^, ^12^, ^13^, ^24^ Only about half of families reported a specific point person or care coordinator.

There are some limitations of the study that warrant discussion. The sample size and distribution obtained from the CFR survey responses are skewed in favor of Pearson syndrome, which is not wholly representative of the population of children with SLSMDS.^1^ This is likely a result of self‐selection because The Champ Foundation was originally founded as an advocacy group for families affected by Pearson Syndrome but aims to include families affected by all SLSMDS in their work. It is also not clear whether this sample of children with mitochondrial disease is representative of other mitochondrial diseases. Given the MCN intends to support all children with mitochondrial disease, future research using these scales and other samples is warranted. More so, many of the families in the CFR are from affluent families.^2^ The logistical and time burdens might be different in a more representative sample and demonstrates a need for future research into low‐income families with SLSMDS and the factor(s) that may prevent them from obtaining a diagnosis or continuing care. Lastly, most of the participants in our study reside in North America and Europe, so it is unclear whether our findings are generalizable to broader international communities.

Mitochondrial disease centers and patient advocacy groups can support families and potentially lessen caregiver burden (Table 4). For example, it may be beneficial for mitochondrial disease centers to provide a “patient navigator” to families. Patient navigators may help facilitate the logistical, cost, and psychological support suggested in Table 4.^28^ For example, they can ensure clear information and resources are shared with families prior to their visit and be a liaison between the family and other services offered within the hospital (e.g., meal vouchers). Given the need to address caregiver burdens for less affluent families, mitochondrial centers and patient navigators can leverage existing resources in their hospitals and incorporate social workers as part of the multidisciplinary team.^29^, ^30^ Finally, there may also be a role for mitochondrial centers to conduct a mental health assessment for caregivers and social work follow‐up, when appropriate. Patient advocacy groups may be able to reduce financial burdens by offering travel assistance to mitochondrial disease centers, connecting newly diagnosed patients to informational resources, and facilitating opportunities to meet and talk to other caregivers of children with the same disorder.^31^

**TABLE 4:** Recommendations for mitochondrial disease centers and patient advocacy groups for reducing caregiver burden.

	Mitochondrial disease centers	Patient advocacy groups
Logistical support	Implementation of a patient navigator for newly diagnosed patients and their families Expedite time for *all* families to establish care Efforts should be made to add additional mitochondrial care centers in geographically diverse locations, including rural and international locations Expand virtual telehealth options Clear information and resources should be provided to all families detailing what can be expected when receiving care from a mitochondrial specialist Support families to determine who is their central coordinating specialist	Provide newly diagnosed families with information about mitochondrial disease centers and other resources immediately after diagnosis Connect interested families with research and/or clinical care coordinators
Cost support	Offer meal or parking vouchers Leverage existing resources within the hospital to reduce cost burdens Engage multidisciplinary team, including social worker to support families	Offer travel grants or financial support for multi‐day trips to mitochondrial care centers, or for care not covered by insurance
Psychological support	Consider adding a caregiver mental health and needs assessment at mitochondrial center visits Help families connect with community organizations that can support children with complex medical needs^30^	Facilitate connections between families, either virtually or in person

## 5 FUTURE DIRECTIONS

This preliminary study on caregiver burden for SLSMDS pediatric patients highlighted potential avenues for future research. For example, while the study describes the burden experienced by caregivers, an in‐depth longitudinal study is needed to further characterize the causal relationship diagnosis of SLSMDS and its associated caregiver burden. Other causal variables of interest and impact on caregiver burden include age, timing of diagnosis, phenotype, and medical complexity. Additionally, future research could examine how differences between clinical institutions, in terms of resources, team composition (physician, nurse, dietician, etc.) affect caregiver reports of burden. Findings might inform recommendations for mitochondrial centers to support both the child and the family.

## 6 CONCLUSION

The present study defines the burden felt by caregivers of children with SLSMDS and is in line with recent literature examining caregiver burden among children with more common variants of mitochondrial disease.^8^ Understanding caregiver burden is important for natural history studies to describe the overall effects of a rare disease.^18^ There is a need for expanded financial and logistical support for families accessing mitochondrial care, and a greater emphasis should be placed on psychological problems faced by caregivers.

## AUTHOR CONTRIBUTIONS

McKenzie Chappell conceived, designed, and wrote the manuscript. Elizabeth Reynolds designed and created the Champ Foundation Registry, conducted all data analyses, created figures and tables, and serves as the guarantor for the article. Sumit Parikh helped develop the PRO surveys and CFR design, verified the PRO surveys, and edited the manuscript.

## CONFLICT OF INTEREST STATEMENT

McKenzie Chappell has no disclosures at this time. Elizabeth Reynolds declares she is the founder of The Champ Foundation, is a volunteer, and has no relevant financial conflicts of interest. Dr. Sumit Parikh serves on the Science Advisory Board of The Champ Foundation and principal investigator on the Champ Foundation Registry.

## INFORMED CONSENT

All procedures followed were in accordance with the ethical standards of the responsible committee on human experimentation (institutional and national) and with the Helsinki Declaration of 1975, as revised in 2000 (5). Informed consent was obtained from all patients for being included in the study. Proof that informed consent was obtained must be available upon request.

## Supporting information


**TABLE S1.** Complete MCN‐PNS results (*N* = 38).
**TABLE S2.** Complete ZBI‐22 results (*N* = 33).

## Data Availability

The datasets generated during and/or analyzed during the current study are available from the corresponding author on reasonable request.
