# Supplementary material for: Understanding the impact of pediatric single large‐scale mtDNA deletion syndromes on caregivers: Burdens and challenges
Source: JIMD Rep. 2023 Aug 3;64(5):375–86. doi: 10.1002/jmd2.12385 (PMC10494495; doi:10.1002/jmd2.12385)
Supplement: Supplementary file 1 — TABLE S1. Complete MCN‐PNS results (N = 38). TABLE S2. Complete ZBI‐22 results (N = 33). [file JMD2-64-375-s001.docx]

Supplemental File 1: Complete MCN-PNS Results (N = 38)

| Item | Response options | N | % |
| --- | --- | --- | --- |
| 1. Please choose a CURRENT or MAIN Mitochondrial Center where you receive your care |  |  |  |
|  | Akron Children’s Hospital | 1 | 3% |
|  | Boston Children’s Hospital | 0 | 0% |
|  | Children’s Hospital of Colorado and University of Colorado | 1 | 3% |
|  | Children’s Hospital of Los Angeles | 0 | 0% |
|  | Children’s Hospital of Philadelphia | 0 | 0% |
|  | Children’s Hospital of Pittsburgh | 7 | 23% |
|  | Children’s Mercy Hospital | 0 | 0% |
|  | Cleveland Clinic | 5 | 17% |
|  | Columbia University Medical Center | 0 | 0% |
|  | George Washington University/Children’s National Health System | 0 | 0% |
|  | Icahn School of Medicine at Mount Sinai | 0 | 0% |
|  | Johns Hopkins University and the Kennedy Krieger Institute | 0 | 0% |
|  | Massachusetts General Hospital | 0 | 0% |
|  | Mayo Clinic | 0 | 0% |
|  | Seattle Children’s Hospital | 0 | 0% |
|  | Stanford University | 0 | 0% |
|  | University of North Carolina | 0 | 0% |
|  | University of California, San Diego | 1 | 3% |
|  | University of California, San Francisco | 0 | 0% |
|  | University of Maryland School of Medicine and Medical System | 0 | 0% |
|  | University of Texas McGovern Medical School | 0 | 0% |
|  | Other | 12 | 50% |
| 2. Is the patient this survey is being completed for: |  |  |  |
|  | Pediatric (Less than 18 years) | 33 | 87% |
|  | Adult (18 or older) | 5 | 13% |
| 3. How did you find your current or main Mitochondrial Center? (select all) |  |  |  |
|  | Physician/care provider referral | 20 | 53% |
|  | Clinic/hospital website | 2 | 5% |
|  | From a friend or individual with mitochondrial disease | 6 | 16% |
|  | Mitochondrial Advocacy Group | 3 | 8% |
|  | Social Media | 2 | 5% |
|  | Other | 8 | 21% |
| 4. How long did it take for you to be referred to a mitochondrial specialist after mitochondrial disease was suspected? |  |  |  |
|  | Less than 6 months | 24 | 63% |
|  | 6-12 months | 4 | 11% |
|  | 1-2 years | 1 | 3% |
|  | 2-3 years | 2 | 5% |
|  | Other | 7 | 18% |
| 5. If you were not immediately referred to a mitochondrial specialist after suspected mitochondrial disease diagnosis, please describe why there was a delay. (select all) |  |  |  |
|  | Primary care provider unaware of mitochondrial disease center | 3 | 8% |
|  | No local mitochondrial disease center available | 14 | 37% |
|  | Cost/insurance coverage | 5 | 13% |
|  | Other | 5 | 12% |
| 6. How long did you have to wait between scheduling the appointment and being seen as a new patient at the Mitochondrial Center? |  |  |  |
|  | 0-2 weeks | 10 | 33% |
|  | 2-4 weeks | 3 | 10% |
|  | 1-2 months | 6 | 20% |
|  | 3-5 months | 5 | 16% |
|  | Greater than 6 months | 6 | 20% |
| 7. Prior to your appointment - were you provided enough information so that you could prepare for your visit, know exactly what to expect at the appointment, and prepare your questions? |  |  |  |
|  | Yes | 20 | 59% |
|  | No | 8 | 24% |
|  | I don't know | 6 | 18% |
| 8. Have you experienced a delay in scheduling a follow-up appointment with your mitochondrial specialist? |  |  |  |
|  | No delay | 26 | 76% |
|  | 1 week | 0 | 0% |
|  | Less than a month | 1 | 3% |
|  | 1-3 months | 1 | 3% |
|  | Greater than 3 months | 6 | 18% |
| 9. How many medical centers/locations do you have to attend to receive all your mitochondrial specialty care? |  |  |  |
|  | 1 | 18 | 53% |
|  | 2-3 | 11 | 32% |
|  | 4-5 | 4 | 12% |
|  | Greater than 5 | 1 | 3% |
| 10. If you have to go to more than one center for care, please describe why: |  | NA | NA |
| 11. How many times per year do you visit your Mitochondrial Center? |  |  |  |
|  | Infrequently, less than once per year | 3 | 10% |
|  | Once per year | 8 | 26% |
|  | 2-3 times per year | 16 | 52% |
|  | 4-5 times per year | 0 | 0% |
|  | >5 times per year | 4 | 13% |
| 12. How do you travel to the Mitochondrial Center? (Select all) |  |  |  |
|  | Car | 26 | 68% |
|  | Airplane | 9 | 24% |
|  | Shuttle service | 1 | 3% |
|  | Bus/train | 4 | 11% |
|  | Other | 0 | 0% |
| 13. How long does it take you to arrive at the Mitochondrial Center from your home (this includes total driving, flying or other transportation time)? |  |  |  |
|  | Less than 1 hour | 8 | 24% |
|  | 1-3 hours | 9 | 27% |
|  | 3-5 hours | 8 | 24% |
|  | 5-10 hours | 6 | 18% |
|  | Greater than 10 hours | 2 | 6% |
| 14. If multiday care is required, where do you stay overnight? (Select all) |  |  |  |
|  | At home | 5 | 13% |
|  | Hotel | 16 | 42% |
|  | Family/friend | 4 | 11% |
|  | Charity/sponsored house (ex. Ronald McDonald House) | 1 | 3% |
|  | Other | 5 | 13% |
| 15. How much do you spend per night for accommodations? |  |  |  |
|  | Not applicable | 14 | 47% |
|  | Less than $50 | 0 | 0% |
|  | $50-$100 | 3 | 10% |
|  | $100-150 | 9 | 30% |
|  | Greater than $150 | 4 | 13% |
| 16. What is the average cost per day for parking onsite when attending Mitochondrial Center? |  |  |  |
|  | Not applicable- parking is free | 11 | 32% |
|  | Less than $5.00 | 4 | 12% |
|  | $5.00-$10.00 | 6 | 18% |
|  | $10.00-$15.00 | 4 | 12% |
|  | Greater than $15.00 | 9 | 26% |
| 17. Does your Mitochondrial Center offer a meal voucher? |  |  |  |
|  | Yes | 5 | 15% |
|  | No | 21 | 62% |
|  | I don't know | 8 | 24% |
| 18. How much money do you spend on meals per day per person when you attend Mitochondrial Center? |  |  |  |
|  | I do not purchase food while attending clinic | 8 | 24% |
|  | Less than $20 | 6 | 18% |
|  | $20-$40 | 12 | 35% |
|  | $40-$60 | 8 | 24% |
|  | Greater than $60 | 0 | 0% |
| 19. On average, how long does it take to complete all of your appointments at the Mitochondrial Center? |  |  |  |
|  | 1 day | 19 | 58% |
|  | 2-3 days | 12 | 36% |
|  | 4-5 days | 2 | 6% |
|  | Greater than 1 week | 0 | 0% |
| 20. Is the Mitochondrial Center you attend considered in-network with your insurance company? |  |  |  |
|  | Yes | 19 | 58% |
|  | No | 10 | 30% |
|  | I don't know | 4 | 12% |
| 21. On a scale of 1-5, how easy is it to understand the medical information your mitochondrial specialists share with you? |  |  |  |
|  | (1) I understand all the information | 15 | 44% |
|  | (2) I understand most of the information | 15 | 44% |
|  | (3) I understand some of the information | 3 | 9% |
|  | (4) I do not understand most of the information | 1 | 3% |
|  | (5) I do not understand any of the information | 0 | 0% |
| 22. How would you prefer to learn medical information related to mitochondrial disease? (Select all) |  |  |  |
|  | Handout/brochure | 6 | 16% |
|  | Website | 11 | 29% |
|  | In-depth discussion with mitochondrial disease physician and/or genetic counselor | 33 | 87% |
|  | Video | 5 | 13% |
|  | Other | 0 | 0% |
| 23. Do you leave your visit with a care plan and next steps from your mitochondrial specialists including how to ask follow-up questions? |  |  |  |
|  | Yes | 29 | 85% |
|  | No | 5 | 15% |
|  | I don't know | 0 | 0% |
| 24. Did the Mitochondrial Center explain clearly what information will be used for clinical care and what information will be used for research? |  |  |  |
|  | Yes | 24 | 73% |
|  | No | 4 | 12% |
|  | I don't know | 5 | 15% |
| 25. How would you like to receive your care plan information? (select all) |  |  |  |
|  | Return visit | 17 | 45% |
|  | Handout/letter | 17 | 45% |
|  | Video follow-up visit/telemedicine | 14 | 37% |
|  | Other | 2 | 5% |
| 26. Do you feel like there is an opportunity to ask all of your questions at each Mitochondrial Center visit? |  |  |  |
|  | Yes | 29 | 85% |
|  | No | 4 | 12% |
|  | I don't know | 1 | 3% |
| 27. Does your mitochondrial medicine care team assist with insurance pre-authorization and denials for genetic testing and mitochondrial supplements? |  |  |  |
|  | Yes | 16 | 47% |
|  | No | 7 | 21% |
|  | I don't know | 11 | 32% |
| 28. Do you have a point person/care coordinator through your Mitochondrial Center? |  |  |  |
|  | Yes | 19 | 56% |
|  | No | 13 | 38% |
|  | I don't know | 2 | 6% |
| 29. Did the mitochondrial clinic explain clearly to what extent they can be involved in your care and what are their limitations? |  |  |  |
|  | Yes | 19 | 59% |
|  | No | 9 | 28% |
|  | I don't know | 4 | 13% |
| 30. Which medical provider do you contact for non-urgent mitochondrial-related medical issues? (select all) |  |  |  |
|  | Primary care provider | 19 | 50% |
|  | Mitochondrial disease physician | 12 | 32% |
|  | Mitochondrial disease care coordinator/nurse | 7 | 18% |
|  | Primary care nurse | 1 | 3% |
|  | Other | 6 | 16% |
| 31. Which medical provider do you contact during a medical emergency? (select all) |  |  |  |
|  | Emergency room provider | 13 | 34% |
|  | Primary care provider | 12 | 32% |
|  | Mitochondrial disease physician | 17 | 45% |
|  | Mitochondrial disease care coordinator/nurse | 2 | 5% |
|  | Primary care nurse | 1 | 3% |
|  | Other | 2 | 5% |
| 32. Has your mitochondrial care team written an emergency letter for you? |  |  |  |
|  | Yes | 20 | 57% |
|  | No | 15 | 43% |
|  | I don't know | 0 | 0% |
| 33. Does your mitochondrial care team communicate with your primary care provider and other specialists (share clinic notes, discuss care/recommendations, etc.)? |  |  |  |
|  | Yes | 27 | 79% |
|  | No | 3 | 9% |
|  | I don't know | 4 | 12% |
| 34. Does your mitochondrial care team address non-medical concerns associated with mitochondrial disease such as social work or psychiatric care? |  |  |  |
|  | Yes | 16 | 47% |
|  | No | 14 | 41% |
|  | I don't know | 4 | 12% |
| 35. Are physician visits a burden on family finances due to copays, parking, food, gas, etc.? |  |  |  |
|  | Yes | 15 | 44% |
|  | No | 15 | 44% |
|  | I don't know | 4 | 12% |
| 36. Are physician visits difficult due to mobility issues? |  |  |  |
|  | Yes | 4 | 12% |
|  | No | 28 | 82% |
|  | I don't know | 2 | 6% |
| 37. How many hospitalizations or emergency room visits have you had in the past year? |  |  |  |
|  | 0 | 7 | 21% |
|  | 1-2 | 11 | 32% |
|  | 3-4 | 13 | 38% |
|  | 5_10 | 3 | 9% |
|  | Greater than 10 | 0 | 0% |
| 38. Do you avoid use of the Emergency Room for any reason? (select all) |  |  |  |
|  | Cost- too expensive | 3 | 8% |
|  | Lack of understanding of mitochondrial disease by ER/ED team | 9 | 24% |
|  | Fear of being exposed to additional illnesses | 14 | 37% |
|  | Too much time | 7 | 18% |
|  | Other | 1 | 3% |
| 39. When discharged from the hospital, do you feel that you have adequate coordination of your medical care including appropriate support services and knowing who to contact with any issues of concerns? |  |  |  |
|  | Yes | 26 | 79% |
|  | No | 5 | 15% |
|  | I don't know | 2 | 6% |
| 40. Please describe concerns or barriers you experience during emergency department visits and/or inpatient hospitalizations. |  | NA | NA |
| 41. How may your emergency department and/or inpatient hospitalization care be improved? |  | NA | NA |
| 42. Since you began care at a Mitochondrial Center, do you feel that the quality of your care has: |  |  |  |
|  | Improved significantly | 10 | 34% |
|  | Improved somewhat | 11 | 38% |
|  | Stayed about the same | 8 | 28% |
|  | Decreased somewhat | 0 | 0% |
|  | Decreased significantly | 0 | 0% |
| 43. How can the mitochondrial care team improve your overall mitochondrial disease care? |  | NA | NA |
| 44. Please describe three items that are most important regarding your mitochondrial disease care. |  | NA | NA |
| 45. Please describe any current gaps or aspects that are missing in your mitochondrial disease care. |  | NA | NA |
| 46. Please select one or more barriers/obstacles that are present in your care for mitochondrial disease. |  |  |  |
|  | No barriers | 4 | 11% |
|  | Distance to travel to Mitochondrial Center | 17 | 45% |
|  | Transportation | 8 | 21% |
|  | Lodging | 8 | 21% |
|  | Childcare | 6 | 16% |
|  | Insurance coverage/cost of appointments | 10 | 26% |
|  | Loss of work/wages | 7 | 18% |
|  | Lack of referral | 2 | 5% |
|  | Lack of necessary medical specialties/services | 8 | 21% |
|  | Lack of care coordination for multisystem disease | 10 | 26% |
|  | Lack of continuity of care between multiple medical specialists | 8 | 21% |
|  | Lack of non-medical support (social, psychological, financial) | 9 | 24% |
|  | Other | 2 | 5% |

Supplemental File 2: Complete ZBI-22 Results (N = 33)

| Item | Response options | N | % |
| --- | --- | --- | --- |
| 1. Do you feel that the affected individual asks for more help than he/she needs? |  |  |  |
|  | Never | 7 | 21% |
|  | Rarely | 9 | 27% |
|  | Sometimes | 12 | 36% |
|  | Quite frequently | 5 | 15% |
|  | Nearly always | 0 | 0% |
| 2. Do you feel that because of the time you spend with the affected individual that you don’t have enough time for yourself? |  |  |  |
|  | Never | 5 | 15% |
|  | Rarely | 2 | 6% |
|  | Sometimes | 14 | 42% |
|  | Quite frequently | 10 | 30% |
|  | Nearly always | 2 | 6% |
| 3. Do you feel stressed between caring for the affected individual and trying to meet other responsibilities for your family or work? |  |  |  |
|  | Never | 2 | 6% |
|  | Rarely | 5 | 15% |
|  | Sometimes | 8 | 24% |
|  | Quite frequently | 11 | 33% |
|  | Nearly always | 7 | 21% |
| 4. Do you feel embarrassed over the affected individual’s behavior? |  |  |  |
|  | Never | 18 | 55% |
|  | Rarely | 7 | 21% |
|  | Sometimes | 6 | 18% |
|  | Quite frequently | 2 | 6% |
|  | Nearly always | 0 | 0% |
| 5. Do you feel angry when you are around the affected individual? |  |  |  |
|  | Never | 16 | 50% |
|  | Rarely | 10 | 31% |
|  | Sometimes | 5 | 16% |
|  | Quite frequently | 1 | 3% |
|  | Nearly always | 0 | 0% |
| 6. Do you feel that the affected individual currently affects your relationship with other family members or friends in a negative way? |  |  |  |
|  | Never | 15 | 46% |
|  | Rarely | 6 | 18% |
|  | Sometimes | 7 | 21% |
|  | Quite frequently | 4 | 12% |
|  | Nearly always | 1 | 3% |
| 7. Are you afraid what the future holds for the affected individual? |  |  |  |
|  | Never | 2 | 6% |
|  | Rarely | 0 | 0% |
|  | Sometimes | 3 | 9% |
|  | Quite frequently | 8 | 24% |
|  | Nearly always | 20 | 61% |
| 8. Do you feel the affected individual is dependent on you? |  |  |  |
|  | Never | 2 | 6% |
|  | Rarely | 1 | 3% |
|  | Sometimes | 5 | 16% |
|  | Quite frequently | 9 | 28% |
|  | Nearly always | 15 | 47% |
| 9. Do you feel strained when you are around the affected individual? |  |  |  |
|  | Never | 10 | 30% |
|  | Rarely | 12 | 36% |
|  | Sometimes | 7 | 21% |
|  | Quite frequently | 4 | 12% |
|  | Nearly always |  |  |
| 10. Do you feel your health has suffered because of your involvement with the affected individual? |  |  |  |
|  | Never | 7 | 21% |
|  | Rarely | 10 | 30% |
|  | Sometimes | 12 | 36% |
|  | Quite frequently | 4 | 12% |
|  | Nearly always | 0 | 0% |
| 11. Do you feel that you don’t have as much privacy as you would like because of the affected individual? |  |  |  |
|  | Never | 15 | 46% |
|  | Rarely | 7 | 21% |
|  | Sometimes | 5 | 15% |
|  | Quite frequently | 5 | 15% |
|  | Nearly always | 1 | 3% |
| 12. Do you feel that your social life has suffered because you are caring for the affected individual? |  |  |  |
|  | Never | 7 | 21% |
|  | Rarely | 6 | 18% |
|  | Sometimes | 11 | 33% |
|  | Quite frequently | 6 | 18% |
|  | Nearly always | 3 | 9% |
| 13. Do you feel uncomfortable about having friends over because of the affected individual? |  |  |  |
|  | Never | 19 | 58% |
|  | Rarely | 7 | 21% |
|  | Sometimes | 6 | 18% |
|  | Quite frequently | 0 | 0% |
|  | Nearly always | 1 | 3% |
| 14. Do you feel that the affected individual seems to expect you to take care of him/her as if you were the only one he/she could depend on? |  |  |  |
|  | Never | 14 | 42% |
|  | Rarely | 6 | 18% |
|  | Sometimes | 8 | 24% |
|  | Quite frequently | 1 | 3% |
|  | Nearly always | 4 | 12% |
| 15. Do you feel that you don’t have enough money to take care of the affected individual in addition to the rest of your expenses? |  |  |  |
|  | Never | 10 | 30% |
|  | Rarely | 6 | 18% |
|  | Sometimes | 6 | 18% |
|  | Quite frequently | 7 | 21% |
|  | Nearly always | 4 | 12% |
| 16. Do you feel that you will be unable to take care of the affected individual much longer? |  |  |  |
|  | Never | 25 | 76% |
|  | Rarely | 7 | 21% |
|  | Sometimes | 1 | 3% |
|  | Quite frequently | 0 | 0% |
|  | Nearly always | 0 | 0% |
| 17. Do you feel like you have lost control of your life since the affected individual’s illness? |  |  |  |
|  | Never | 8 | 24% |
|  | Rarely | 12 | 36% |
|  | Sometimes | 6 | 18% |
|  | Quite frequently | 7 | 21% |
|  | Nearly always | 0 | 0% |
| 18. Do you wish you could leave the care of your affected individual to someone else? |  |  |  |
|  | Never | 24 | 73% |
|  | Rarely | 4 | 12% |
|  | Sometimes | 4 | 12% |
|  | Quite frequently | 1 | 3% |
|  | Nearly always | 0 | 0% |
| 19. Do you feel uncertain about what to do about the affected individual? |  |  |  |
|  | Never | 10 | 30% |
|  | Rarely | 5 | 15% |
|  | Sometimes | 11 | 33% |
|  | Quite frequently | 5 | 15% |
|  | Nearly always | 2 | 6% |
| 20. Do you feel like you should be doing more for the affected individual? |  |  |  |
|  | Never | 1 | 3% |
|  | Rarely | 3 | 10% |
|  | Sometimes | 18 | 58% |
|  | Quite frequently | 5 | 16% |
|  | Nearly always | 4 | 13% |
| 21. Do you feel like you could do a better job in caring for the affected individual? |  |  |  |
|  | Never | 3 | 9% |
|  | Rarely | 15 | 46% |
|  | Sometimes | 8 | 24% |
|  | Quite frequently | 5 | 15% |
|  | Nearly always | 2 | 6% |
| 22. Overall, how burdened do you feel in caring for the affected individual? |  |  |  |
|  | Never | 6 | 18% |
|  | Rarely | 9 | 27% |
|  | Sometimes | 7 | 21% |
|  | Quite frequently | 7 | 21% |
|  | Nearly always | 4 | 12% |
